# Lipofuscin: The Missing Link in the Worsening of Obstructive Sleep Apnea

**DOI:** 10.1002/lary.70344

**Published:** 2025-12-27

**Authors:** Kristine Fahl, Carlos Modesto Vera Palomino, Mauricio da Silva Baptista, Adriano Polican Ciena, Danielle Andrade da Silva Dantas, Luiz Ubirajara Sennes, Marucia Chacur, Michel Burihan Cahali

**Affiliations:** ^1^ Department of Otolaryngology, Hospital das Clinicas HCFMUSP, Faculdade de Medicina Universidade de Sao Paulo Sao Paulo Brazil; ^2^ Department of Biochemistry, Institute of Chemistry Universidade de Sao Paulo Sao Paulo Brazil; ^3^ Laboratory of Morphology and Physical Activity (LAMAF) Institute of Biosciences, Sao Paulo State University (UNESP) Rio Claro Brazil; ^4^ Department of Otolaryngology. Faculdade de Medicina Universidade Federal de Pernambuco Recife Brazil; ^5^ Laboratory of Neuroanatomy Functional of Pain, Departamento de Anatomia, Institute of Biomedical Science Universidade de Sao Paulo Sao Paulo Brazil

**Keywords:** disease aggravation, lipofuscin, obstructive sleep apnea, oxidative stress, upper airway muscles

## Abstract

**Objectives:**

There is a lack of evidence linking oxidative stress to muscle dysfunction and the worsening of obstructive sleep apnea (OSA). Lipofuscin is a permanent intracellular deposit that accumulates with aging or oxidative stress. Its increase in skeletal muscles may lead to limitations in muscle function, such as weakness and fatigue. We aim to investigate whether lipofuscin deposits in the upper airway muscles of patients with severe OSA are increased compared to those in patients with primary snoring or very mild OSA.

**Methods:**

We studied pharyngeal framework muscle tissue samples from non‐obese adults, with one group having severe OSA and another having primary snoring or very mild OSA (snorer group). Lipofuscin was identified by analyzing staining patterns from Sudan Black B and fluorescence microscopy. We compared lipofuscin levels and distribution in the muscle fibers between the groups.

**Results:**

Despite presenting similar age and body mass index, patients with severe OSA exhibited markedly larger and more concentrated deposits of lipofuscin in the upper airway muscles, particularly along the borders of the muscle fascicles, compared to the snorer group.

**Conclusion:**

Compared to primary snorers, patients with severe OSA accumulate lipofuscin in their pharyngeal muscles. Our findings may provide novel insight into the role of lipofuscin as both a marker and a cause of the aggravation of OSA, in light of a possible lipofuscin‐induced progressive myopathy.

**Level of Evidence:**

3.

## Graphical abstract

Lipofuscin, also known as the age pigment, is a permanent autofluorescent intracellular deposit that accumulates with aging or oxidative stress, thereby interfering with cellular function. This study revealed significantly larger deposits of lipofuscin in the lateral pharyngeal wall muscles of patients with severe obstructive sleep apnea (OSA) compared to an age‐ and body mass index‐matched snorer group. This novel finding illuminates a potential connection between oxidative stress, upper airway muscle dysfunction, and the worsening of OSA.

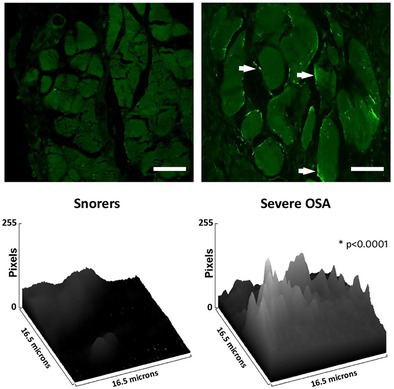

## 1 Introduction

The physiological basis for the exacerbation of obstructive sleep apnea (OSA), independent of weight gain, is poorly understood [1, 2]. Older individuals, even with a similar body mass index (BMI) to younger individuals, may experience more severe OSA due to fat infiltration in the upper airway and visceral areas [3]. Anatomically, hyoid position, tongue volume, and pharyngeal length show moderate correlation with upper airway collapsibility [4]. Functionally, patients with OSA demonstrate an inability of the upper airway muscles to protect pharyngeal patency during sleep, which is referred to as neuromuscular dysfunction. The role of muscles in this trait has been a topic of debate [5, 6].

Compared to patients without OSA, the pharyngeal muscles in OSA show increased fibrosis (which is also associated with aging), which may increase stiffness, reduce airway compliance, and interfere with the mechanical coupling between muscle fibers and surrounding tissues [7]. In addition, the palatopharyngeus muscle in OSA seems to have a portion of its fibers shifted from slow‐twitch to fast‐twitch fibers, which are more prone to fatigue [8]. This muscle also shows possible signs of reinnervation following nerve injury [9, 10]. These findings suggest that OSA may affect the function of pharyngeal muscles. However, these tissue changes do not seem to reflect oxidative stress, which is a hallmark of OSA, because they are believed to result from mechanical injuries during sleep, either due to repeated collapses of the upper airway or persistent heavy snoring.

A clear footprint of OSA reflecting progressive upper airway muscle dysfunction linked to oxidative stress is still missing. The palatoglossus muscle expresses increased protein carbonyls (a marker of oxidative stress) in severe OSA compared to mild OSA [11] and the palatopharyngeus muscle shows reduced mitochondrial activity in OSA compared to controls [12]. Nevertheless, the external intercostal muscle in OSA shows increased levels of oxidative stress markers and reduced endurance, and inexplicably, both fail to improve with continuous positive airway pressure treatment [13], suggesting a hidden, irreversible pathogenesis.

Lipofuscin is an intracellular deposit that accumulates with aging or oxidative stress. It is composed of highly oxidized and covalently bound proteins, lipids, metal ions, and sugar residues [14, 15, 16]. Mitochondria that are damaged by oxidative stress are the main contributors to lipofuscin formation [17]. All these highly oxidized and cross‐linked materials cannot be degraded in lysosomes or exocytosed from the cell (lysosomes are the cell's waste disposal units), so they become permanent intralysosomal aggregates within the cytoplasm [17, 18, 19, 20]. The build‐up of this often‐called age pigment takes up space within the cell and potentially impairs lysosomal function and interferes with cellular processes [21]. Most tissues handle this issue by splitting lipofuscin content during cell division into daughter cells. However, this option is not available in cells such as neurons, cardiac myocytes, and skeletal muscle fibers [22].

Being a complex pigment, lipofuscin detection requires a combination of methods for precise identification. Sudan Black B is a dye that specifically binds to lipids. In addition, lipofuscin granules have a characteristic autofluorescence that can be observed under certain wavelengths of light, making them detectable with fluorescence microscopy. Although other cellular components may also exhibit autofluorescence, pairing fluorescence with specific staining techniques can confirm the presence of lipofuscin.

During life, lipofuscin accumulates in a nearly linear manner, whereas its rate of formation can increase in the final stages of senescence or in the progression of several pathological processes [23]. Its increase leads to impaired energy production and utilization within muscle fibers. As a consequence, related signs and symptoms include muscle weakness, pain, and fatigue [24]. We speculate that, as a consequence of intermittent hypoxia and oxidative stress, lipofuscin build‐up in the upper airway muscles may play an important role in the aggravation of OSA, regardless of weight gain or aging, providing sought‐after evidence for the link between oxidative stress and upper airway muscle dysfunction. To our knowledge, lipofuscin has never been evaluated in the upper airway of patients with OSA.

## 2 Materials and Methods

This study was approved by our Institutional Review Board (protocol number 965.007/2015 and 3.121.150/2019) and registered in the Brazilian National Unified Database for Human Research, No. CAAE 36571514.4.0000.0068. The original collection of our specimens was approved by our Institutional Review Board (protocol number 559/2005), for which all patients provided written informed consent.

We analyzed formalin‐fixed paraffin‐embedded blocks of deep pharyngeal muscle tissues removed during surgeries to treat OSA or chronic primary snoring, performed in our institution between 2005 and 2006. These muscle samples were collected in a previous study designed to evaluate the role of extracellular matrix composition in the pathogenesis of OSA [7]. Briefly, after the surgeries, the cold dissected tissues were fixed in 10% buffered formalin for 24 h and then processed and paraffin‐embedded.

### 2.1 Study Population

Pharyngeal muscle samples came from 13 non‐obese patients (BMI < 30 kg/m^2^, age, 20–54 years). These samples were selected based on remaining muscle tissue in blocks for analysis, to compare two very distinct groups: one with severe OSA and one with primary heavy snoring or very mild OSA (hereafter we refer to this group as Snorers). The exclusion criteria were neuromuscular diseases, retroglossal obstructions, non‐controlled or controlled for less than 1 year hypothyroidism, previous oropharyngeal surgeries, Down syndrome, craniofacial deformities, and history of phlegmon or peritonsillar abscesses. All patients had been tested with in‐lab type I polysomnography within the 3 months prior to surgery. All polysomnograms and surgeries were performed in the absence of acute upper airway inflammation, and hypopneas were scored according to the criteria of that period (> 50% decrease in airflow or a clear decrease in airflow inferior to 50% associated with either at least a 4% oxygen desaturation or an arousal, lasting 10 s or longer) [25]. All patients presented with chronic habitual heavy snoring. We divided the patients into a Severe OSA group (*n* = 6), who presented excessive daytime sleepiness [26] and an apnea‐hypopnea index, AHI > 30 events/h, and a Snorer group (*n* = 7), who did not have excessive daytime sleepiness [26] and had an almost normal AHI (≤ 6 events/h).

### 2.2 Tissue Preparation and Image Analysis

We studied sections of muscle fascicles from the lateral pharyngeal wall (palatopharyngeus and superior pharyngeal constrictor). The paraffin‐embedded blocks were cut into 5 μm‐thick sections. In order to avoid confusing lipofuscin with other pigments (like ceroids), we employed a three‐step methodology to identify it: Hematoxylin and Eosin, Sudan Black B, and Fluorescence. During tissue preparation and image analysis, examiners were blind to the patient's group. All reagents used were of analytical grade.

#### 2.2.1 Hematoxylin and Eosin

We used standard deparaffinization and rehydration protocol for the slices. After 45 s of Eosin staining, the samples were rinsed and then incubated in Hematoxylin for 2 min (Hematoxylin and Eosin stains, Sigma‐Aldrich, St. Louis, MO, USA). The dry slides were then mounted under a microscope (DMi8 model, Leica Microsystems, Germany) equipped with a camera (DMC6200 model, Leica Microsystems, Germany) with a 40× magnification. This analysis served as a first scan for the presence and location of lipid‐like granules in the muscle fascicles, which might represent lipofuscin accumulation. To quantify those granules, we used the image processing software ImageJ (version 2.14.0/1.54f, Windows 11, Fiji, open‐source project) to create 8‐bit grayscale images, with 255 representing no stain (absolute white) and 0 representing absolute black (maximum staining), and thresholds ranging from 150 to 0 were considered indicative of the presence of lipofuscins. For each patient, we analyzed five square areas of muscle, each measuring 16.5 × 16.5 μm. The number of pixels stained per 16.5 × 16.5 μm blocks—within the grayscale range from 150 to 0—was compared between groups.

#### 2.2.2 Sudan Black B

A carefully placed drop of a 0.3% Sudan Black B solution (catalog# 199664, Sigma‐Aldrich, St. Louis, MO, USA) on the slide helps to prevent the precipitation of Sudan Black B in the tissues. After washing the mixture using 70% ethanol and distilled water, the staining outcome turned out as intended in 30 min [27]. Lipofuscin‐like accumulation staining was then visualized with the same Leica DMi8 microscope, using a 40× objective in the bright‐field mode. Using the same image processing software ImageJ, we created histograms from differences in staining across the groups, using 8‐bit grayscale images, with pixel intensity values ranging from 0 (black) to 255 (white), thresholds ranging from 150 to 0 indicating the presence of lipofuscins, and darkness correlating to lipofuscin concentration. Sudan Black B staining analysis utilized the full range of available values: for each patient, we analyzed five square areas of muscle, each measuring 16.5 × 16.5 μm. All stained pixels in these areas, according to the grayscale values, were used to generate histograms for each group. The number of pixels stained per 16.5 × 16.5 μm blocks—within the range from 150 to 0—was compared between groups.

#### 2.2.3 Fluorescence Microscopy

Deparaffinized and rehydrated, the tissue sections were ready for fluorescence microscopy. FITC is a fluorescent dye that is excited by blue light and emits green light. Lipofuscin autofluorescence was detected using a FITC filter cube (Ex 488/40; dichroic 505; Em 527/30: Excitation wavelength 488 nm with bandwidth 40 nm, which allows light from 460 to 500 nm to pass through; dichroic mirror 505 nm, which redirects wavelengths below 505 nm towards the sample and allows wavelengths above 505 nm to pass through to the detection system; Emission wavelength 527 nm with bandwidth 30 nm) and maximized signal collection imaging using the Leica DMRA2 Fluorescence Microscope with a 40× magnification and a Leica DFC350FX digital camera for fluorescence detection (Leica Microsystems, Germany). The detection of lipofuscin fluorescence after excitation at 488 nm was followed as previously described [28, 29]. On each patient, we analyzed fluorescence in five square areas of muscle measuring 16.5 × 16.5 μm each. We used the image processing software ImageJ to create histograms (using 8‐bit grayscale images) representing mean fluorescence intensity per unit of area analyzed in each group. Higher fluorescence intensity values indicate greater lipofuscin accumulation. Zero represents no fluorescence (black in the image) and 255 means maximum fluorescence (brightest white). Images were analyzed to calculate the percent area positive for fluorescence and this was compared between groups.

### 2.3 Statistical Analysis

To compare the demographic and clinical characteristics of the groups, we used the Mann–Whitney *U* test and Fisher's Exact Test. To compare the histological data between the two groups, we performed the Mann–Whitney *U* test. Data were summarized as the mean (median) and interquartile range. We used the GraphPad Prism 7 software for statistical analysis (GraphPad Software, San Diego, CA, USA). Statistical significance was considered at *p* < 0.05.

## 3 Results

The demographic and clinical characteristics of the groups are shown in Table 1. One patient in the Snorer group had hypertension, while another was a smoker. Patients in the severe OSA group did not present any other comorbidities. Age and BMI were not significantly different between groups.

**TABLE 1:** Demographic, anthropometric and polysomnographic data of the groups.

Characteristic	Snorers	Severe OSA	*p*
Cases, *n*	7	6	
Sex (male/female), *n*	5/2	6/0	0.46^†^
Age, years	42.3 (43.0) [36.0–49.5]	33.8 (31.0) [21.5–45.8]	0.25
AHI, events/h	3.6 (3.0) [2.0–5.1]	44.6 (38.8) [33.1–46.3]	0.003*
Minimum SpO_2_, %	86.4 (88.0) [81.5–90.5]	77.8 (80.5) [72.0–86.0]	0.09
BMI, kg/m^2^	24.5 (24.7) [24.3–25.0]	24.1 (23.5) [22.9–24.4]	0.13

*Note*: Data are presented as mean (median) [interquartile range].

Abbreviations: AHI: apnea‐hypopnea index; BMI: body mass index; OSA: obstructive sleep apnea; SpO_2_: oxyhemoglobin saturation.

^†^ Fisher's Exact test.

* *p* < 0.05. Mann–Whitney *U* test.

**FIGURE 1:**
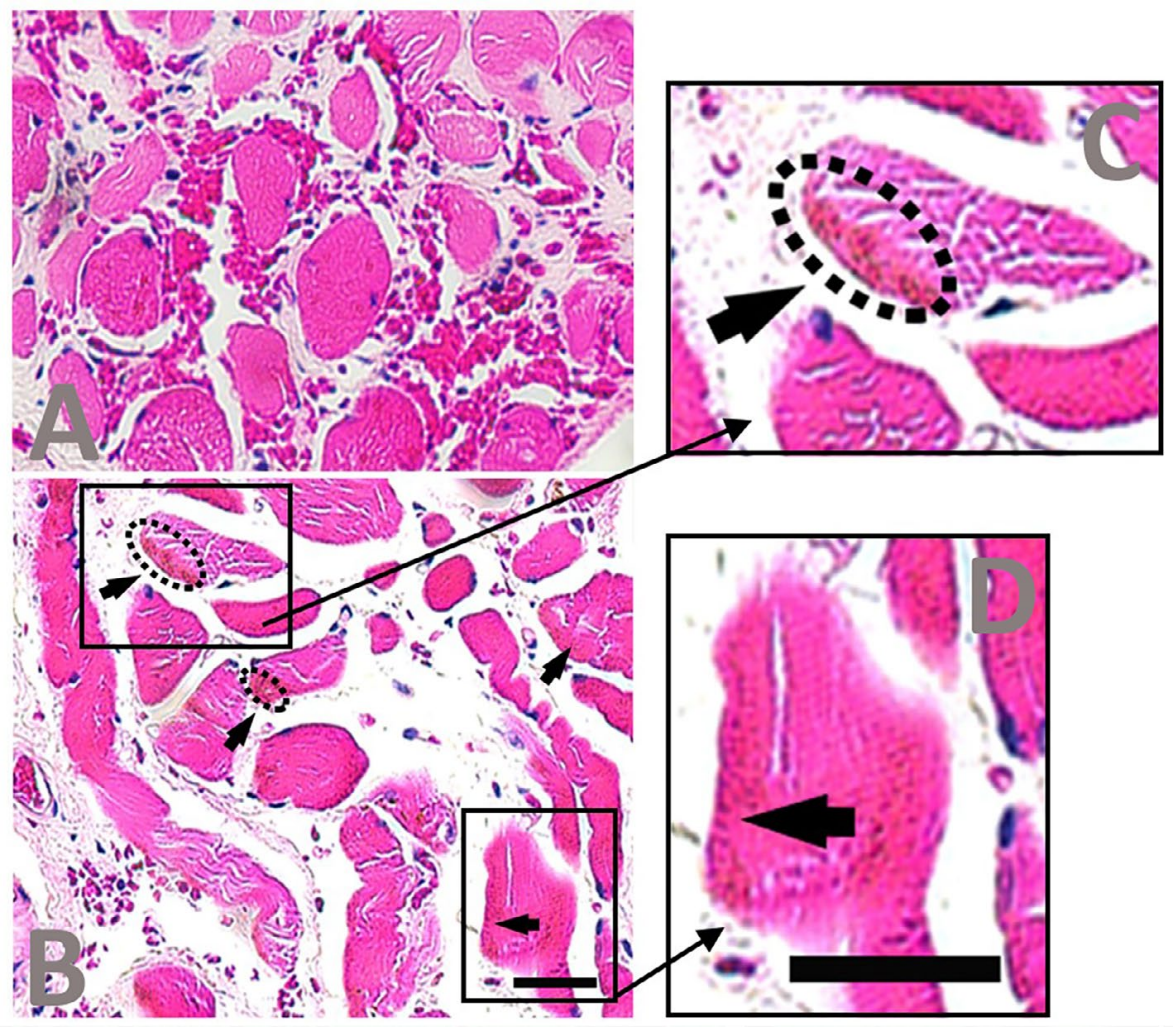
Illustrative sections of pharyngeal muscles stained with Hematoxylin and Eosin. A primary snoring patient presenting no suggestive evidence of lipofuscin accumulation (A). A severe OSA patient showing small, grouped lipid‐like granules within the muscle fascicles (arrows), suggesting the area of interest to investigate lipofuscin accumulation (B, zooming in C and D). Scale bar: 30 μm. Objective lens: 40×.

In the Hematoxylin and Eosin staining, muscle fascicles in the Snorer group presented homogeneous staining and no suggestive evidence of lipofuscin accumulation. By contrast, the Severe OSA group showed small, round, lipid‐like granules that were grouped at the peripheral regions of many muscle fascicles, indicating possible lipofuscin accumulation (Figure 1). The measurements on the grayscale‐converted images confirmed the accumulation of these granules in the muscle periphery of the Severe OSA group. The number of pixels stained (with intensities from 0 to 150) within a 16.5 × 16.5 μm area showed a mean (median) [interquartile range] of 2.3 (2.0) [2.0–2.8] in the Snorer group and 10.6 (10.0) [9.0–11.8] in the Severe OSA group (*p* = 0.005).

**FIGURE 2:**
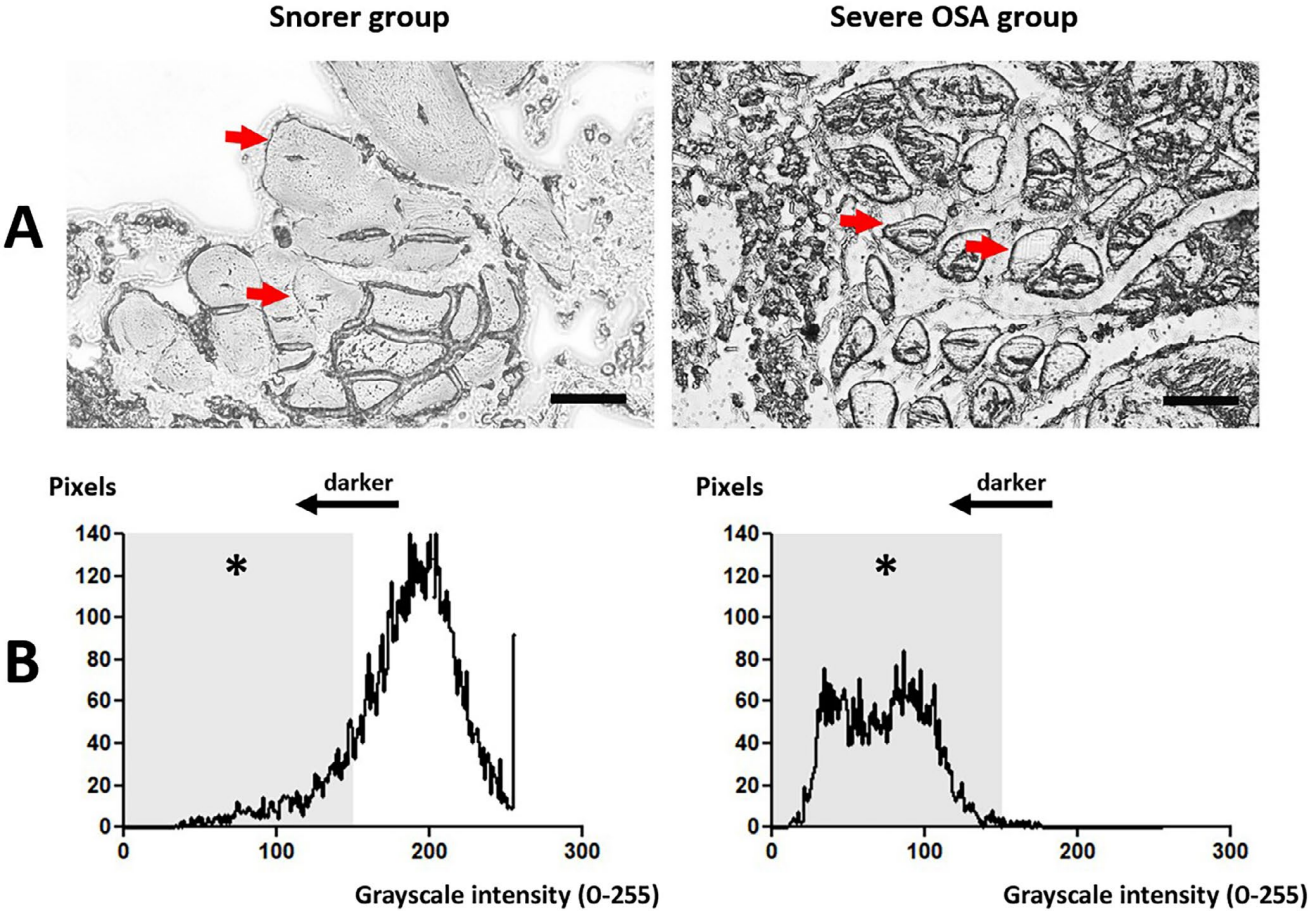
Illustrative sections of pharyngeal muscles stained with Sudan Black B, representing the Snorer group and the Severe OSA group. The stain accumulates along the border of the muscle fascicles (red arrows), appearing darker in the Severe OSA group (panel A). Histograms showing the distribution of the pixels according to the grayscale intensity of the stain in each group (0 = absolute black, 255 = absolute white). The shaded area represents the grayscale range associated with lipofuscin (0 to 150, darker correlates with higher lipofuscin concentration) (panel B). * The number of pixels stained in the shaded area was significantly greater in the Severe OSA group (*p* < 0.0001). Scale bar: 30 μm. Objective lens: 40×.

Sudan Black B staining typically accumulated along the border of the muscle fascicles, presenting a higher intensity (darker) in the Severe OSA group (Figure 2). The intensity histograms show that, unlike the Snorer group, the vast majority of pixels stained with Sudan Black B presented intensities suggestive of lipofuscin (ranging from 0 to 150, Figure 2). The mean (median) and [interquartile range] of the number of pixels stained in the area of 16.5 × 16.5 μm (within the intensities from 0 to 150) were 30.6 (31.5) [24.3–35.0] in the Snorer group and 296.3 (284.5) [271.0–332.0] in the Severe OSA group (*p* < 0.0001). Both the stained area and intensity of the Sudan Black B stain suggest a predominant presence of lipofuscin in the Severe OSA group, matching the location of the lipid‐like granules observed in the Hematoxylin and Eosin staining.

**FIGURE 3:**
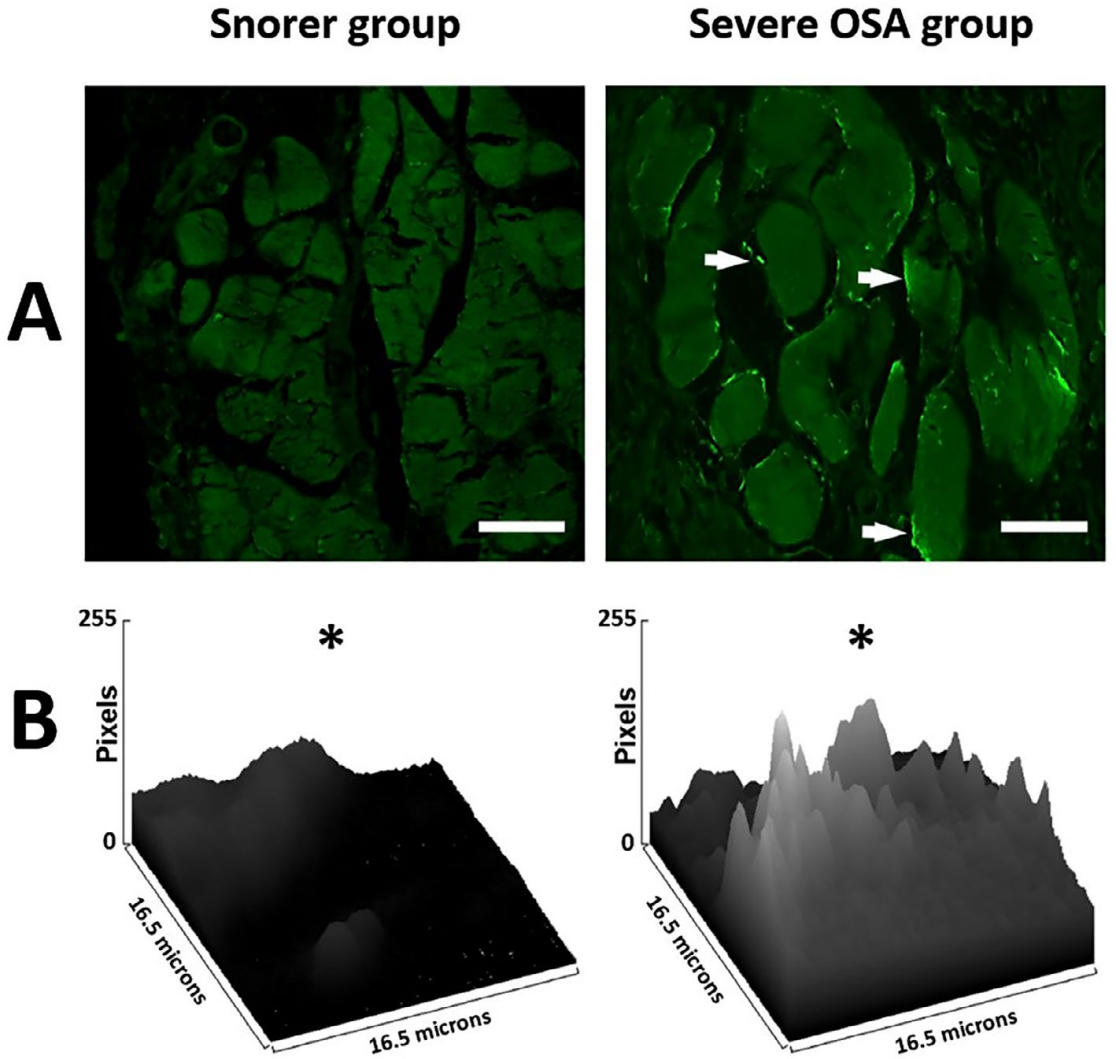
Illustrative sections of pharyngeal muscles under Fluorescence microscopy to detect Lipofuscin autofluorescence (Excitation wavelength 488 nm, Emission wavelength 527 nm), representing the Snorer group and the Severe OSA group. Fluorescence accumulates along the border of the muscle fascicles (white arrows), with higher intensity in the Severe OSA group (panel A). Histograms showing mean fluorescence intensity per unit of area analyzed in each group (0 = no fluorescence, 255 = maximum fluorescence) (panel B). Higher fluorescence intensity values indicate greater lipofuscin accumulation. * The percentage area of the tissue exhibiting fluorescence was significantly greater in the Severe OSA group (*p* < 0.0001). Scale bar: 30 μm. Objective lens: 40×.

Fluorescence microscopy demonstrated higher autofluorescence intensity along the border of the muscle fascicles in the Severe OSA group (Figure 3), suggesting lipofuscin accumulation at the same location identified with Hematoxylin and Eosin and with Sudan Black B staining. The histograms of mean fluorescence intensity per unit of area analyzed in each group show higher peaks (meaning more lipofuscin) in the Severe OSA group (Figure 3). The mean (median) and [interquartile range] of the percentage area of the tissue exhibiting fluorescence, per 16.5 × 16.5 μm blocks, were 21.3 (21.4) [17.4–23.8] in the Snorer group and 56.1 (59.0) [45.0–62.6] in the Severe OSA group (*p* < 0.0001).

## 4 Discussion

Our study revealed that patients with severe OSA exhibit significantly higher lipofuscin accumulation in the upper airway muscles compared to patients with primary snoring or very mild OSA (Snorer group). Lipofuscin was typically found to accumulate along the peripheral regions of the muscle fascicles and was identified with high certainty using a combination of two separate stains: Sudan Black B and fluorescence microscopy. Given that an increase in lipofuscin may lead to limitations in muscle function, this study provides evidence of a possible link between upper airway damage and the worsening of OSA not attributable to aging or weight gain.

The severity of OSA has been associated with increased levels of oxidative stress markers in the serum, upper and lower airway [5, 11, 13, 30, 31]. Mitochondria are both a major source and target of reactive oxygen species, which, in turn, can impair mitochondrial function, contributing to muscle weakness and fatigue [24, 32]. Nevertheless, it seems that a clearer link between the consequences of oxidative stress and the worsening of OSA is missing. Given that lipofuscin is a permanent and progressive material that accumulates mainly as a consequence of aging or oxidative stress, our finding of an increase in lipofuscin in upper airway muscles in non‐obese patients with severe OSA, compared to age‐ and BMI‐matched snorers, points to a possible pathophysiological explanation for the worsening of OSA. Our samples were taken from deeper pharyngeal layers, which constitute the pharyngeal framework and do not seem to express increased oxidative stress markers or accumulation of inflammatory cells according to OSA severity [7, 33]. This observation may be explained by the relatively short half‐life of many markers triggered by intermittent hypoxia, as the extended period of normal breathing prior to tissue harvesting would allow for their clearance [33]. This is in contrast to the findings in superficial pharyngeal layers, which seem to be more affected by snoring vibrations and pharyngeal pressure swings due to repetitive apneas [34]. Our study particularly excluded the confounder of obesity, as there is evidence that inflammatory markers in the soft palate are linked to obesity, rather than to OSA [35]. The possible role of lipofuscin as both a marker and a cause of the aggravation of OSA deserves further studies. Given the cross‐sectional nature of our study, we couldn't identify the initiating pathways for lipofuscin accumulation in the upper airway muscles. It seems logical to assume an interplay between OSA and lipofuscin leading to a self‐perpetuating cycle that worsens OSA; however, it remains unclear which factor initiates this cycle. While OSA induces oxidative stress that may increase lipofuscin levels, it is also plausible that a primary tissue deficiency in antioxidant activity could promote oxidative stress, thereby increase lipofuscins and contribute to OSA [34].

There is previous evidence of a lipid‐related content at the upper airway worsening OSA, regardless of weight gain [3, 36]. It appears to be fat infiltration in the upper airway of older individuals with more severe OSA [3] and also an increased amount of lipid droplets in pharyngeal muscles that correlates with OSA severity [36]. We believe our findings of lipofuscin in severe OSA corroborate these studies. The apparent concentration of lipofuscin at the periphery of muscle fascicles appears to be a novel observation. One possible explanation is that type‐I fibers, known to be rich in mitochondria, may be more prevalent in these regions and thus provide a localized source for lipofuscin. Further research is required to validate this hypothesis.

In skeletal muscle fibers, lipofuscin progressively accumulates through life [23]. Oxidative stress increases lipofuscin deposits, which can disrupt normal cellular processes and promote cellular dysfunction and aging [14]. This process has been highlighted in patients with conditions like Alzheimer's disease, Parkinson's disease, and chronic obstructive pulmonary disease [14, 37]. In the muscle fibers, lipofuscin aggregates can exacerbate mitochondrial abnormalities, as seen in mitochondrial myopathy, contributing to muscle weakness [38, 39, 40]. It can also lead to muscle atrophy and reduced regenerative capacity [41]. We suggest that the lipofuscin build‐up we found in upper airway muscles in young non‐obese adults (mean age, 33.8 years) with severe OSA is a likely explanation for the worsening of OSA, thus supporting the role of upper airway myopathy in the pathophysiology of OSA [6]. Presumably, the impact of this process would not be limited to sleep: this lipofuscin build‐up may also play a role in the frequent finding of swallowing abnormalities detected in examinations of patients with OSA [42].

As a pilot investigation, we acknowledge the limitations of our study. Our sample size is limited, not only due to the availability of tissue samples from previous work but also because we focused on non‐obese patients from very distinct spectrums of OSA within the same age range. Also, there were only two women in our study and they belonged to the Snorer group. Future studies including obese, older patients, more women, and cases with moderate OSA are needed to explore the role of lipofuscin in OSA aggravation on those groups. While we assume that OSA severity implies greater oxidative stress, future investigations on lipofuscin should also measure alternative oxidative stress markers to better understand the triad of oxidative stress, lipofuscin, and worsening OSA. Unfortunately, the historical nature of the cohort limited our polysomnographic analysis to AHI and minimum oxyhemoglobin saturation, metrics that inadequately reflect intermittent hypoxia compared to more comprehensive measures like the hypoxic burden or oxygen desaturation index. Finally, future studies should also correlate evidence of upper airway muscle dysfunction with lipofuscin deposits in these tissues.

## 5 Conclusion

There is a significant increase in lipofuscin deposits in pharyngeal muscle fibers in patients with severe OSA compared to those with primary snoring or very mild OSA. Generated by oxidative stress and linked to muscle dysfunction, lipofuscin emerges as a possible cause for the aggravation of OSA.

## Funding

This work was supported by Fundação de Amparo à Pesquisa do Estado de São Paulo, 2020/09089‐8.

## Conflicts of Interest

Luiz Ubirajara Sennes and Michel Burihan Cahali: Stockholders in the Brazilian company Biologix that markets a home sleep apnea test (not used in this study). The other authors declare no conflicts of interest.

## Data Availability

The data that support the findings of this study are available on request from the corresponding author. The data are not publicly available due to privacy or ethical restrictions.
